# Modulation of SIRT-1, NF-κB/TNF-α/IL-6, and ERK/Caspase-3 by Lutein Mitigates Methotrexate-Induced Hepatotoxicity

**DOI:** 10.3390/ph18121787

**Published:** 2025-11-24

**Authors:** Areej M. Abdelwahab, Heba A. Habib, Mostafa A. Darwish, Yousef A. Bin Jardan, Gehan H. Heeba

**Affiliations:** 1 Department of Pharmacology and Toxicology, Faculty of Pharmacy, Sphinx University, New Assuit City 71515, Egypt; areej.atya@sphinx.edu.eg (A.M.A.); mostafa.darwesh@sphinx.edu.eg; 2 Department of Pharmacology and Toxicology, Faculty of Pharmacy, Minia University, Minia 61519, Egypt; 3 Department of Pharmaceutics, College of Pharmacy, King Saud University, Riyadh 11451, Saudi Arabia; ybinjardan@ksu.edu.sa

**Keywords:** MTX, lutein, NF-κB, SIRT-1, inflammation, oxidative stress

## Abstract

**Background/Objectives**: Methotrexate (MTX) is an effective immunosuppressant and chemotherapeutic agent used to manage various cancers and inflammatory illnesses, but its hepatotoxic hazards pose a major challenge to its clinical application. The preventive influence of lutein against MTX-elicited liver damage was assessed in the present study, focusing on its impact on the SIRT-1, NF-κB -TNF-α-IL-6, and ERK-caspase-3 signaling pathways. **Methods**: Forty male Wistar rats were randomly assigned into control, Lutein 100 mg, MTX, MTX + Lutein 50 mg, and MTX + Lutein 100 mg groups. MTX groups were intraperitoneally injected with MTX (40 mg/kg) on day 7, while lutein was daily administered by oral route for 10 days. **Results**: MTX-induced liver damage was evident by significant structural damage and elevation in hepatic biochemical markers. MTX intoxication induced disturbance in the oxidant/antioxidant equilibrium, downregulated SIRT-1, inflammation reflected by upregulation of pro-inflammatory cytokines, NF-κB, eventual TNF-α and IL-6 levels, and apoptosis evident by elevated ERK-caspase-3 in hepatic tissue. Notably, pre-conditioning with lutein mitigated these harmful effects. Lutein’s therapeutic impact is mechanistically explained by its antioxidant potential, in addition to its ability to enhance SIRT-1 expression and abrogate the inflammatory NF-κB -TNF-α-IL-6 pathway and apoptotic ERK-caspase-3 response. **Conclusions**: Overall, these outcomes suggest that lutein could be applied as a promising therapeutic tool to be combined with MTX to attenuate the risk of its hepatotoxicity.

## 1. Introduction

Methotrexate (MTX) is a widely used chemotherapeutic agent that is applied for managing multiple types of malignancies [1]. It is a folate derivative that principally inhibits dihydrofolate reductase (DHFR), eventually inhibiting synthesis and repair of DNA as well as cellular division, specifically in cells with high proliferation rates [2]. Its uses vary depending on doses. At low doses, it is commonly applied for managing conditions of autoimmune and inflammatory insult due to its immunomodulatory and anti-inflammatory properties [3,4]. At high doses, it is used for controlling various malignancies [5,6]. Despite its clinical efficacy, MTX is associated with several adverse effects harming the gastrointestinal tract, liver, lung, and kidney, which can limit its long-term use [7,8]. MTX’s hepatotoxicity, such as cirrhosis as well as fibrosis, occurs in approximately twenty-six and fifty percent of MTX administers [9,10].

Although MTX is known to induce toxicity in the liver, the precise mechanism underlying this toxicity remains not fully understood [11,12]. Folylpolyglutamate synthetase (FPGS) in hepatocytes transforms MTX into MTX polyglutamates (MTX-PGs), resulting in oxidative stress within the liver [13]. The hepatic folate level is restricted by MTX-PGs, which, in turn, block the synthesis of proteins, particular amino acids, and nucleic acids, causing structural damage, impairing liver function, enzyme leakage, and ultimately cellular apoptosis [14,15].

Sirtuin-1 (SIRT-1), a NAD^+^ dependent deacetylase, plays a critical role in regulating hepatic cellular homeostasis and protecting against hepatotoxicity. The inverse relation between SIRT-1 and oxidative stress, inflammation, and apoptosis pathways has been confirmed [16,17] due to its ability to deacetylate and inhibit inflammatory markers, thereby reducing inflammatory responses, while also enhancing antioxidant defenses by activating pathways like nuclear factor erythroid 2-related factor 2 (Nrf2). SIRT-1 downregulation, leading to oxidative damage and eventual inflammatory and apoptotic cascades, has been extensively reported in the context of liver impairment [18,19,20,21]. Thus, SIRT-1 modulation is considered a promising therapeutic strategy to mitigate hepatotoxicity induced by various toxins and chemotherapeutic agents [22].

Lutein, a naturally occurring carotenoid, is widely found in green leafy vegetables, egg yolk, and certain fruits [23]. It is vital for preserving eye health by accumulating in the macula and retina, filtering harmful blue light, and neutralizing reactive oxygen species (ROS) [24,25]. Beyond its ophthalmoprotective effects, lutein has recently shown potential benefits in cognitive health [26] and cardiovascular protection [27]. Of interest is that the hepatoprotective effect of lutein has also been demonstrated in various experimental models of liver dysfunction triggered by carbon tetrachloride (CCl_4_), paracetamol, ethanol [28], diclofenac [29], chronic alcohol intake [30], non-alcoholic fatty liver disease (NAFLD) [31], and paraquat [32], discussing its antioxidant and anti-inflammatory impact. Nevertheless, its role in the prevention and management of MTX-produced liver illness is not reported.

In light of the published reports, this study aimed to evaluate, among first attempts, the ameliorative effects of lutein against MTX-induced hepatotoxicity at different doses. Additionally, the possible signaling pathways involved in these protective impacts were investigated in terms of SIRT-1, nuclear factor kappa-B (NF-κB), tumor necrosis factor-α (TNF-α), interleukin-6 (IL-6), and extracellular signal-regulated kinases (ERK)-caspase-3.

## 2. Results

### 2.1. Effect of MTX and/or Lutein on Markers of Hepatic Performance

Following the MTX intoxication, the MTX group tended to increase relative hepatic weight, while the two MTX groups pretreated with lutein tended to decrease this parameter. However, no significant difference can be observed among the five groups involved in the study, as shown in Table 1.

Meanwhile, MTX treatment led to a significant (*p* < 0.05) deterioration in liver function as indicated by high levels of serum alanine aminotransferase (ALT) and aspartate aminotransferase (AST) compared to the control group. However, these markers were significantly (*p* < 0.05) lowered in groups treated with low and high doses of lutein, as illustrated in Table 1.

Remarkably, MTX-intoxicated rats treated with a high dose of lutein showed a more pronounced decline in serum ALT and AST levels relative to the group subjected to a low dose of lutein, as shown in Table 1.

### 2.2. Effect of MTX and/or Lutein on Hepatic Histopathological Features

**Figure 1:**
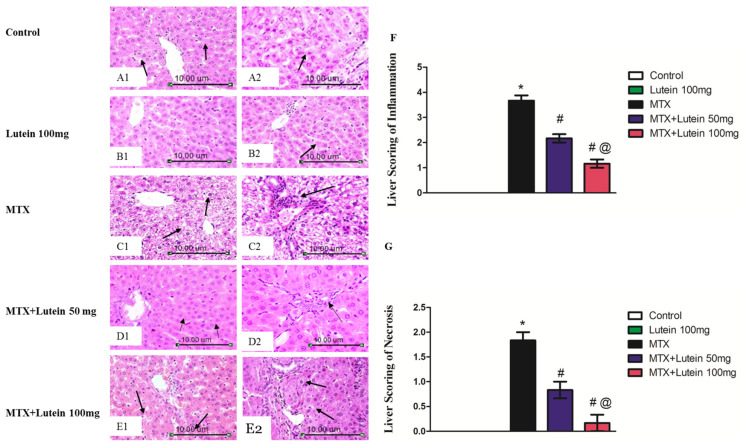
Effect of MTX and/or lutein at two different doses on hematoxylin and eosin (H and E)-stained hepatic sections. Photomicrographs of the liver tissue sections from control group ((**A1**), X200 and (**A2**), X400) and Lutein 100 mg group ((**B1**), X200 and (**B2**), X400)) exhibited a histological structure that appeared normal. The MTX group shows focal necrosis and hydropic degeneration of hepatocytes (**C1**, X200, arrow) and a notable increase in the portal lymphocyte number (**C2**, X400, arrow). The MTX + Lutein 50 mg group shows swelling of hepatocytes and few numbers of apoptotic cells (**D1**, X200, arrow) and portal lymphocytes (**D2**, X200, arrow). The MTX + Lutein 100 mg group shows normal organization of hepatic cords with hyperplasia of Kupffer cells (**E1**, X200, arrow) and swelling of hepatocytes with narrowing of sinusoids in the peripheral zone (**E2**, X200, arrow). (**F**,**G**) show the scoring of inflammation and necrosis, respectively, in hepatocyte of each group. Data are represented as mean ± SEM. * Significant different compared to the control group, # significant different compared to the MTX group, and @ significant different compared to the MTX + Lutein 50 mg group at *p* < 0.05. MTX: Methotrexate.

Figure 1(A1,A2,B1,B2) demonstrate sections of liver tissue from the control and lutein 100 mg groups. The tissue sections revealed well-preserved hepatic lobules with healthy hepatocytes organized in radiating plates around the central vein. The hepatocytes displayed prominent round nuclei and eosinophilic cytoplasm, preserving normal cellular morphology. The sinusoids were lined by fenestrated endothelial cells, along with a normal distribution of Kupffer cells. The portal area, including the bile duct, portal vein, and hepatic artery, presented a normal histological structure without any evidence of inflammation or damage.

In contrast, sections of hepatic tissue from the MTX group showed disrupted hepatic structure with disorganized hepatic cords and degenerative changes in hepatocytes. These changes included focal necrosis, hydropic degeneration, and a few microvesicular steatosis and apoptotic bodies. There was also a significant rise in portal lymphocytes, reflecting marked inflammation (Figure 1(C1,C2)).

Meanwhile, MTX + Lutein 50 mg revealed hepatocellular swelling and a limited number of apoptotic cells. Hepatic sinusoids appeared narrowed, accompanied by Kupffer cell hyperplasia. A discreet increase in the number of portal lymphocytes was noticed (Figure 1(D1,D2)).

Notably, the hepatic tissue sections from the MTX + Lutein 100 mg showed normal organization of hepatic cords with hyperplasia of Kupffer cells. The hepatocytes in the peripheral zone showed mild swelling and narrowing of hepatic sinusoids. The number of portal lymphocytes remained normal (Figure 1(E1,E2)).

Figure 1F,G showed a bar chart of histopathological scoring of inflammation and necrosis in all studied groups, revealing that the intoxication of MTX induced significant necrosis and inflammation. However, pretreatment with either a low dose or a high dose of lutein showed obvious improvements in MTX-induced hepatic damage with more pronounced improvement achieved by high dose.

### 2.3. Effect of MTX and/or Lutein on Hepatic Oxidative Status

When compared with the control group, a single dose of MTX (40 mg/kg, I.P.) led to a significant (*p* < 0.05) increase in malondialdehyde (MDA) level in hepatic homogenate. Additionally, administration of MTX led to a marked decrease in reduced glutathione (GSH) level and superoxide dismutase (SOD) activity relative to the control group.

**Figure 2:**
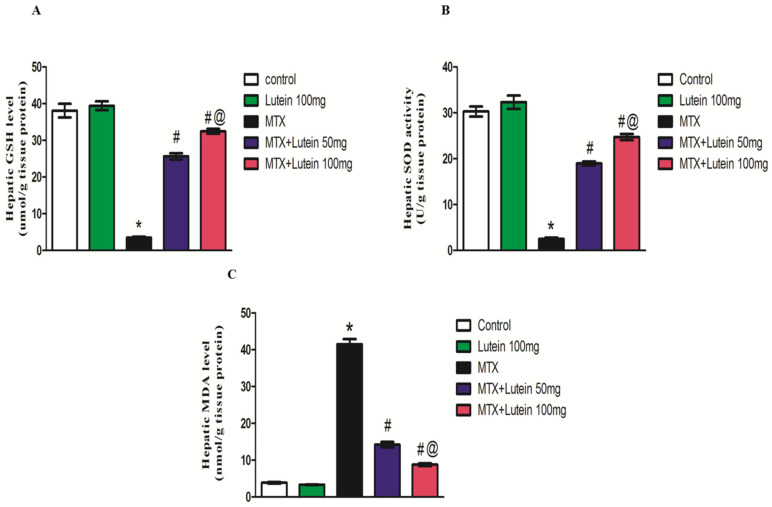
Effect of MTX and/or lutein at two different doses on hepatic GSH (**A**), SOD (**B**), and MDA (**C**). All data points are shown as the means ± SEM (*n* = 6–8). * Significant different compared to the control group, # significant different compared to the MTX group, and @ significant different compared to the MTX + Lutein 50 mg group at *p* < 0.05. MTX: Methotrexate, GSH: Reduced glutathione, SOD: Superoxide dismutase, and MDA: Malondialdehyde.

On the other hand, concomitant treatment with lutein at different doses (50 and 100 mg/kg, given I.G.) significantly (*p* < 0.05) alleviated the MTX-elicited increase in MDA level. Both doses also restored the GSH content and SOD activity compared to the MTX-treated group, as illustrated in Figure 2. It is worth noticing that the higher dose of lutein demonstrated greater antioxidant potential, represented by a more pronounced reduction in MDA level and elevation in antioxidant defensive tools, GSH and SOD, in comparison to the lower dose.

### 2.4. Effect of MTX and/or Lutein on Expression of SIRT-1 in Hepatic Tissue

In comparison with the control group, MTX (40 mg/kg, I.P.) challenge showed a significant suppression of SIRT-1 expression in the investigated tissues. Interestingly, prior treatment with lutein, either at low or high dose, significantly (*p* < 0.05) mitigated MTX-induced downregulation in SIRT-1 expression when compared to rats that were treated with MTX alone.

**Figure 3:**
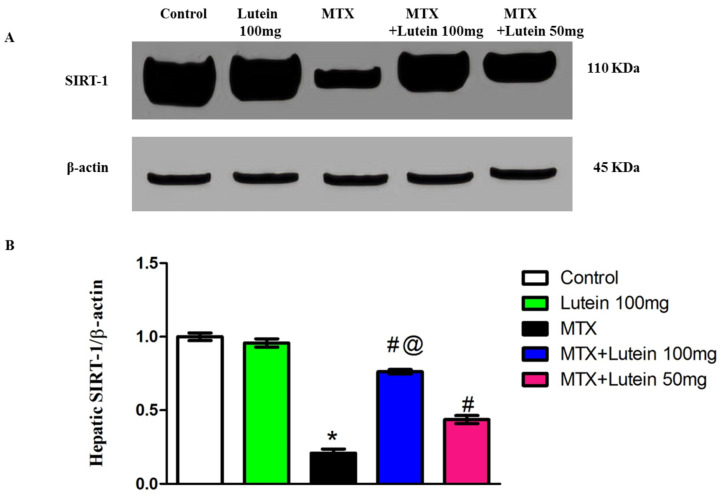
Effect of MTX and/or lutein at two different doses on hepatic SIRT-1 protein expression. (**A**) Representative Western blots showing target, SIRT-1, protein bands from each experimental group. (**B**) Bar graphs represent the quantified densitometric analysis of hepatic SIRT-1 protein expression. The value for each bar is represented as mean ± SEM. * Significant different compared to the control group, # significant different compared to the MTX group, and @ significant different compared to the MTX + Lutein 50 mg group at *p* < 0.05. MTX: Methotrexate, SIRT-1: Sirtuin-1.

Moreover, enhancement of SIRT-1 expression in hepatic tissues was significantly (*p* < 0.05) more observable in the MTX rats subjected to preconditioning with the higher dose (100 mg/kg) compared to those treated with the lower dose (50 mg/kg) (Figure 3).

### 2.5. Effect of MTX and/or Lutein on Expression of NF-κB in Hepatic Tissue

**Figure 4:**
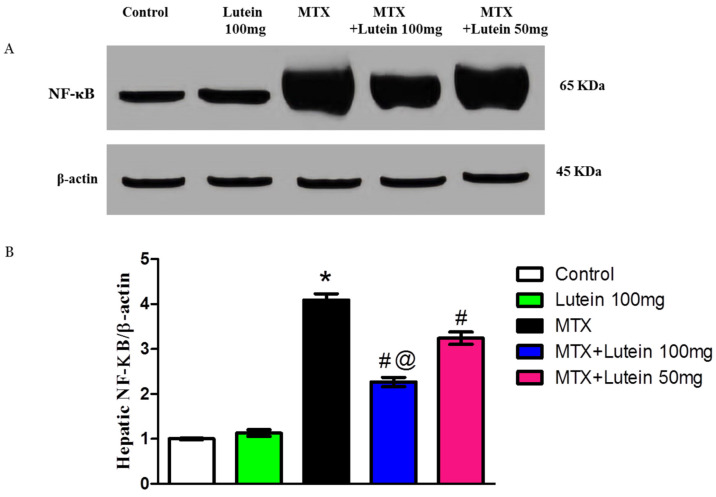
Effect of MTX and/or lutein at two different doses on hepatic NF-κB protein expression. (**A**) Western blot representations illustrating NF-κB protein bands for each group. (**B**) Bar graphs represent the quantified densitometric analysis of hepatic expression of NF-κB protein. The value for each bar is represented as mean ± SEM. * Significant different compared to the control group, # significant different compared to the MTX group, and @ significant different compared to the MTX + Lutein 50 mg group at *p* < 0.05.MTX: Methotrexate, NF-κB: Nuclear factor kappa-B.

As illustrated in Figure 4, protein expression of NF-κB in the liver was significantly (*p* < 0.05) increased in MTX group compared to control animals. Moreover, administration of lutein at low and high doses I.G. significantly (*p* < 0.05) suppressed this elevation induced by MTX. In addition, lutein co-treatment with MTX resulted in a dose-related reduction in NF-κB expression.

The group that received 100 mg/kg lutein demonstrated a more substantial reduction in NF-κB expression than the group that received 50 mg/kg lutein.

### 2.6. Effect of MTX and/or Lutein on TNF-α and IL-6 in Hepatic Tissue

Methotrexate (MTX) induces inflammation, which is one of the most important pathways that contribute to its toxicity. Therefore, levels of TNF-α and IL-6 proteins were tested as inflammatory biomarkers. Similar to the NF-κB pattern, a significant (*p* < 0.05) increase in TNF-α and IL-6 protein levels was observed in the MTX-intoxicated animals compared to the control group. Intriguingly, lutein administration at both examined doses significantly (*p* < 0.05) attenuated MTX-provoked robust elevation in TNF-α and IL-6 levels as compared to the group challenged with MTX alone.

**Figure 5:**
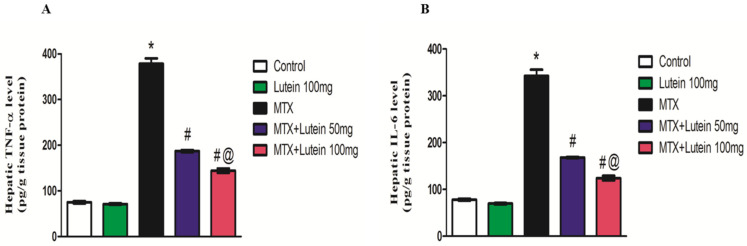
Effect of MTX and/or lutein at two different doses on hepatic TNF-α (**A**) and IL-6 levels (**B**). All data points are shown as the means ± SEM (*n* = 6). * Significant different compared to the control group, # significant different compared to the MTX group, and @ significant different compared to the MTX + Lutein 50 mg group at *p* < 0.05. MTX: Methotrexate, TNF-α: Tumor necrosis factors, IL-6: Interleukin-6.

The reduction in TNF-α and IL-6 was dose-related; the higher dose of lutein provided a further attenuation in the aforementioned inflammatory markers as shown in Figure 5.

### 2.7. Effect of MTX and/or Lutein on Gene Expression and Levels of ERK and Caspase-3 in Hepatic Tissue

**Figure 6:**
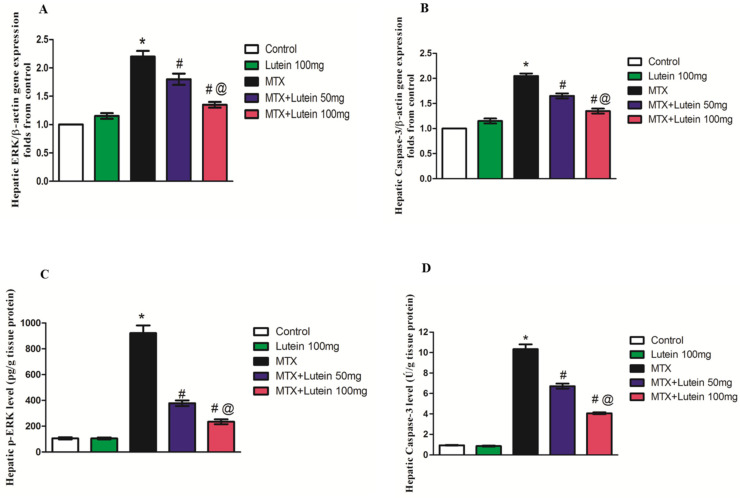
Effects of lutein (50 and 100 mg/kg, I.G.) on hepatic ERK (**A**) and caspase-3 (**B**) gene expression and hepatic level of p-ERK (**C**) and caspase-3 (**D**) in a rodent model of MTX-induced hepatotoxicity. All data points are shown as the means ± SEM. * Significant different compared to the control group, # significant different compared to the MTX group, and @ significant different compared to the MTX + Lutein 50 mg group at *p* < 0.05. MTX: Methotrexate, ERK: Extracellular signal-regulated kinases.

A significant (*p* < 0.05) increase in ERK and caspase-3 mRNA expression and their levels was found in the hepatic tissues of MTX-injected animals in comparison with normal rats, as shown by quantitative real-time polymerase chain reaction (qRT-PCR) analyses and the ELISA technique. Treatment with the two different investigated doses of lutein before MTX injection caused a remarkable decline in the mRNA expression and level of ERK and caspase-3 when compared with the MTX-injected rats. Furthermore, the decline in hepatic ERK and caspase-3 was more pronounced in MTX rats treated with 100 mg/kg of lutein than in the MTX + lutein 50 mg group, as presented in Figure 6.

## 3. Discussion

Methotrexate (MTX) is a commonly used antineoplastic and immunosuppressant drug; however, its application is often clinically limited due to the associated hazards, particularly hepatic injury [8]. This study reports the inhibitory effect of lutein on MTX-induced hepatotoxicity in rats, as presented here by restoration of liver performance and preservation of intact liver structure. This protective effect might be mediated by SIRT-1 modulation and antagonizing oxidative damage and inflammatory and apoptotic responses.

Liver injury occurred here after MTX intoxication presented in the form of elevated ALT and AST values, which are indicators of liver dysfunction [14] and a remarkable destruction of hepatic tissue, observed upon histological examination: disorganization of hepatic cords, focal necrosis, and hydropic degeneration of hepatocytes with hepatic apoptosis. These observations are aligned with prior studies [33,34,35,36]. Degenerated hepatocytes and loss of the integrity of hepatocyte structure with subsequent leakage of its intracellular contents underlie the noticed increase in the liver enzymes [37,38].

It is worth mentioning that lutein pretreatment at both used doses provided hepatoprotection, verified by a significant attenuation in MTX-induced elevated ALT and AST concentrations and noticeable preservation of hepatic architecture; reduced hepatocellular degeneration and minimized inflammatory cell infiltration are consistent with earlier studies reporting the lutein hepatoprotective influence [28,29,30,32]. Of interest, disturbance in liver enzymes and histological alteration were significantly attenuated in the MTX + Lutein 100 mg group compared to MTX-challenged rats treated with a lower dose, as seen in the experiment carried out by Edward, Ajibade [32]. These outcomes spotlight the possible effect of lutein in hindering liver impairment induced by MTX. In the context of these observations, we dug deeper to explore the implicated molecular mechanisms that can explain the hepatoprotection of lutein in the MTX setting.

Although the exact mechanisms of MTX-induced liver dysfunction remain unclear, increased hepatic MTX-PGs are embroiled in MTX-elicited hepatic injury due to eventual reduction in folic acid, which, in turn, leads to multiple harmful events; impaired equilibrium between oxidant/antioxidant factors besides inflammatory and apoptotic responses [14].

Oxidative damage is remarkably engaged with MTX hepatic impairment [9,14,39]. MTX disrupts mitochondrial DNA, resulting in mitochondrial dysfunction, which consequently induces excessive ROS generation [40,41]. The robust increase in ROS damages protein and DNA and peroxidizes membrane lipid. Lipid peroxidation disarranges the membrane, receptors, and enzymes, which alters membrane permeability, illustrating the observed increase in ALT and AST [14,42].

These reported findings align with the results observed in this study, where MTX administration led to an obvious increase in MDA content, a lipid peroxidation indicator, and a remarkable decline in the protective antioxidant tools, GSH as well as SOD activity, in hepatic tissue [43]. MTX inhibits NADPH, which is important for maintaining glutathione reductase, which explains the noted attenuation in GSH [44].

N-acetylcysteine and silymarin are known hepatoprotective therapeutic approaches through battling oxidative stress [45,46,47]. Protection exerted by N-acetylcysteine is mediated by its rapid deacetylation to cysteine, the rate-limiting substrate for hepatic GSH synthesis, thereby restoring the liver’s intrinsic detoxification capacity [48]. Through free radical scavenging, enhanced antioxidant enzyme activity, stabilization of cell membrane, and prevention of lipid peroxidation, silymarin provided hepatoprotection in various liver disorders [49].

Here, lutein could significantly restore the balance of the oxidative condition, indicated by decreased MDA, in addition to increased GSH and SOD. This protective antioxidant activity was afforded by the two examined lutein doses, but 100 mg produced a more pronounced antioxidant effect. The lutein antioxidant impact was reported in various experimental investigations, including age-related macular degeneration [50], some cardiovascular and neurological disorders like Alzheimer’s, Parkinson’s, and age-related cognitive impairment [51,52]. Additionally, in vitro experiments confirmed that lutein possesses free radical scavenging activity by inhibiting the production of ROS [53].

Regarding liver injury, lutein could protect against cisplatin [54]- and NAFLD [31]-induced hepatotoxicity by significantly lowering hepatic MDA levels and enhancing total antioxidant capacity. Zeaxanthin, another carotenoid, showed a potential preservative effect in an experimental rodent model of NAFLD mediated by antagonizing liver oxidative stress [55].

Furthermore, there is a strong interplay between MTX hepatotoxicity and SIRT-1 downregulation [18,19], as observed in this study. SIRT-1 produces hepatoprotection via various protective mechanisms, antagonizing inflammatory pathways, oxidative cellular damage and apoptosis [16,17]. It is worth mentioning that SIRT-1-mediated Nrf2, a master organizer of antioxidant response, deacetylation boosts the antioxidant response, increasing hepatocellular resilience against oxidative stress [56]. Of interest, Wang, Wang [57] demonstrated that lutein activates the SIRT-1 pathway. Huang, Duan [58] reported the connection between amelioration of Parkinson’s disease and SIRT-1 activation. Here, the inverse relation between SIRT-1 and oxidative stress was mirrored by reduced SIRT-1 expression and distorted oxidative balance in the MTX group. However, the reduction in hepatic SIRT-1 expression was alleviated by lutein treatment at doses of either 50 mg/kg or 100 mg/kg, with more pronounced alleviation observed in rats pretreated with a high dose. Therefore, we propose that MTX-induced oxidative stress may be mediated by the suppression of SIRT-1 expression and targeting SIRT-1 may be a prospective tool to dampen MTX-related hepatic illnesses.

Additionally, the contribution of inflammation in MTX-induced development of liver injury was documented [59,60]. MTX intoxication leads to an increase in NF-κB and TNF-α in liver tissues, suggesting a connection between these factors and MTX-induced functional impairment [59,60]. NF-κB signaling activity is heightened by a variety of pro-inflammatory cytokines, such as TNF-α, and its activation triggers the production of numerous genes that code for pro-inflammatory mediators such as TNF-α, IL-6, and IL-1β. This interaction between NF-κB and TNF-α has been previously documented [60].

Furthermore, there is a notable link between NF-κB, SIRT-1, and inflammation. SIRT-1 exerts anti-inflammatory effects by either preventing NF-κB translocation and activation or directly downregulating pro-inflammatory cytokine genes [56,61]. Parallel to reports discussing this link, the liver tissues of rats intoxicated by MTX showed low expression of SIRT-1, along with NF-κB/TNF-α/IL-6 upregulation.

Besides lutein’s antioxidant activity, it has been documented to possess anti-inflammatory properties, indicating its role in counteracting inflammation and oxidative stress associated with various disease states [62]. The current study examines how co-treatment with lutein provides hepatoprotection through reducing the elevated expression of NF-κB/TNF-α/IL-6 due to MTX challenge. These findings are consistent with previous studies that have reported lutein’s anti-inflammatory impacts in diverse contexts, including neurodegenerative disorders and cardiovascular disease [27,58,63]. Moreover, lutein effectively counteracts MTX-induced lung damage by decreasing oxidative stress, inflammation, and apoptosis [64]. Aktas, Gur [54] also reported the inhibitory effect of lutein against cisplatin hepatic impairment by downregulation of TNF-α. Astaxanthin, zeaxanthin’s metabolite, exhibited an organ-protective effect in experimental model of a high fructose diet through modulation of the SIRT-1/NF-κB pathway alongside mitigation of oxidative stress [65].

The observed protective effect of lutein on MTX-induced liver damage in this study appears to be mediated by its ability to modulate the SIRT-1/NF-κB/TNF-α/IL-6 signaling pathway, depending on the dosage used.

To gain a more comprehensive understanding of lutein’s protective effects against MTX-induced hepatic apoptosis, we examined its effects on the expression and levels of key markers of programmed cell death, specifically ERK and caspase-3. Our findings were in agreement with earlier studies [12,14] reporting that MTX-PGs triggered caspase-3 overexpression in liver tissue. This overexpression may be linked to exaggerated ROS generation and increased levels of pro-inflammatory cytokines. The intoxicated group exhibited a significant elevation in these markers mediating the apoptotic pathway in hepatic tissues [66].

The anti-apoptotic influence of lutein has been previously documented against apoptosis induced by glutamate in an in vitro study [67]. Moreover, Chucair, Rotstein [68] highlighted lutein’s ability to hinder apoptosis, protecting photoreceptors. The influence of lutein on ERK and caspase-3 has been investigated in the setting of MTX-induced liver injury. Our findings notably show that lutein treatment significantly reduces the levels of both ERK and caspase-3 when compared to MTX treatment alone. These findings emphasize the anti-apoptotic and cytoprotective properties of lutein, which are facilitated by its significant impact on essential signaling pathways.

Moreover, the ERK/caspase-3, NF-κB and downregulated SIRT-1 pathways are known to interact closely, forming a complex network that controls inflammation, apoptosis, and cell survival [69]. These pathological cascades are reported in hepatic impairment produced by MTX [14,18,19]. These findings suggest that therapeutically manipulating one might indirectly influence the others and mitigate the harmful effects on the liver [21,70]. Therefore, lutein reduces NF-κB/TNF-α/IL-6 and promotes SIRT-1 and mitigates apoptosis, as evidenced by attenuated ERK/caspase-3.

Importantly, clinical studies investigating the anticancer properties of lutein have suggested its possible safeguarding role, especially in the prevention and slowing of the progression of certain cancers. Epidemiological studies suggest that increased dietary intake or higher blood concentrations of lutein are linked to a lower risk of developing cancers, including breast, colon, and lung cancer [71,72]. In cell culture studies, lutein induced cell cycle arrest and promoted programmed cell death in cancer cells without significantly affecting normal cells, exhibiting a selective cytotoxic effect. It also lessened oxidative DNA damage and modulated the expression of crucial regulatory proteins such as Bcl-2-associated X protein (Bax), B-cell lymphoma 2 (Bcl-2), and caspases [73].

In animal models, lutein supplementation led to shrinkage of tumors, as well as reduction in angiogenesis and metastasis, likely by the inhibition of pathways such as NF-κB and mitogen-activated protein kinase (MAPK) that are playing a role in inflammation and injury [74].

Overall, this study provides evidence of lutein’s hepatoprotective effect against MTX-related toxicity, acting through its antioxidant, anti-inflammatory, and anti-apoptotic properties. Lutein enhanced SIRT-1 expression, lowered MDA levels, increased SOD activity and GSH content, inhibited NF-κB/TNF-α/IL-6/signaling, and reduced caspase-3 and ERK expression in liver tissue, resulting in improved liver function markers and histological structure. Lutein, as a natural compound, may be a hopeful tool to lessen the prevalence and seriousness of MTX-related side effects, ultimately improving patient outcomes. However, further clinical research is mandatory to validate these findings in humans and to identify the effectiveness and safety of lutein to mitigate the toxic effects of anticancer drugs.

However, a comparison with N-acetylcysteine as a positive control hepatoprotective agent to benchmark lutein’s efficacy can be conducted. Mechanistic studies to further substantiate the causality of lutein’s protective effect in MTX hepatotoxicity are needed; the need to investigate direct SIRT-1 activation, mitochondrial function, and using pharmacological inhibitors of SIRT-1, such as EX-527, or genetic knockdown, besides pharmacokinetic investigations to precisely correlate lutein tissue levels with the observed therapeutic outcomes are considered limitations of this work that should be focused on in future studies. Moreover, inclusion of both female and male sexes can be performed in the future research to elucidate sex-based differences in MTX toxicity and antioxidant metabolism.

## 4. Materials and Methods

### 4.1. Drugs

Methotrexate (MTX) and lutein were bought from Hikma Pharma (Cairo, Egypt) and from NOW FOODS (Bloomingdale, DuPage County, IL, USA), respectively.

### 4.2. Animals

From Animal Care Unit at Faculty of Pharmacy, Nahda University, 40 adult male *Wistar* rats with weights between 180 and 220 g were utilized. Animals were placed in polypropylene cages for a two-week acclimation period, during which they were kept under standard conditions, including a 12-h light/dark cycle and an adjusted temperature of 25 ± 2 °C. Throughout the study, they had unrestricted access to a standard chow diet and fresh water.

### 4.3. Experimental Design and Induction of Hepatotoxicity

An independent investigator randomly divided the rats into five groups, with each group comprising 8 rats. Eight animals per group were selected based on previous studies [13,75,76,77] conducted in comparable experimental models.

1-Control group: The rats were given corn oil (the vehicle for lutein) by I.G. route for 10 successive days.

2-Lutein 100 mg group: The rats were administered lutein at a dosage of 100 mg/kg/day via I.G. route for 10 days.

3-MTX group: The rats received corn oil intragastrically for 10 successive days and were given a single I.P. MTX injection (40 mg/kg) on day 7.

4-MTX + Lutein 50 mg group: These rats received a low dose (50 mg/kg/day, I.G.) of lutein for 10 successive days and were also administered a single I.P. dose of MTX (40 mg/kg) on the 7th day of the experimentation.

5-MTX + Lutein 100 mg group: Rats were, daily, given a high dose (100 mg/kg, I.G.) of lutein for 10 consecutive days. On day 7, rats of this group were exposed to a single I.P. dose of MTX (40 mg/kg).

The MTX dosing strategy was adopted from methods used in previous studies [60,75,78] reporting the incidence of hepatotoxicity, while that of lutein is based on studies showing its ability to provide organ protection [32,54,79] and also on the preliminary trials conducted in this study.

### 4.4. Blood and Tissue Sampling

A day after the final dose was administered, the rats were anesthetized using pentobarbital sodium (50 mg/kg, I.P.) following the measurement of their body weight. Blood samples were then collected by cardiac puncture into clean centrifuge tubes and centrifuged for 10 min at 3500 rpm to separate the serum. Fresh serum samples were then immediately used for evaluating the status of liver function, including ALT and AST concentration. Parts from the liver were preserved in 10% formalin for histopathological examination, while the rest of the liver tissues were immediately frozen in liquid nitrogen and kept at −80 °C. For biochemical analysis, the liver samples were homogenized in ice-cold phosphate-buffered solution (PBS), centrifuged at 10,000 rpm for 10 min, and the supernatant was subsequently collected for further analysis.

### 4.5. Determination of Liver Function Markers

Levels of serum ALT and AST were used to evaluate liver function. Spectrophotometric determination of ALT and AST levels was performed using commercially available BioMed kits, Cairo, Egypt, based on the method outlined by Henry [80].

### 4.6. Liver Histopathology

Liver specimens were fixed in 10% neutral buffered formalin after being trimmed, rinsed, and dehydrated using graded alcohols, cleared in xylene, and then embedded in paraffin wax. The blocks were then sectioned into 4–6 µm thick slices and stained with H and E stain, adhering to the protocol outlined in reference [81]. The slides were then examined and visualized by an independent examiner who was blinded to experimental findings under a light microscope at 200× and 400× magnification. The histopathological evaluation included scoring of inflammatory changes according to Ramos, Sá [82] and that of necrosis in hepatocytes according to the METAVIR scoring system [83].

### 4.7. Assessment of Oxidative Status

A hallmark of lipid peroxidation, MDA, serves as a marker of oxidative stress. Its concentration was quantified using the thiobarbituric acid reactive species method outlined by Uchiyama and Mihara [84]. Thiobarbituric acid, in an acidic medium, reacts with MDA, forming a pink product spectrophotometrically measured at 534 nm. With the use of 1,1,3,3-tetramethoxypropane standard curve, the recorded absorbance represents the corresponding MDA concentration.

In addition, antioxidant defenses, specifically GSH and SOD, were also analyzed. GSH levels were measured following the protocol proposed by Ellman [85]. The sulfhydryl group present in GSH reducing Ellman’s reagent forms a product having a yellow color which can be detected at 412 nm.

Meanwhile, SOD activity was evaluated using the approach developed by Marklund [86]. Marklund’s [86] method is based on the ability of SOD to prevent autoxidation of pyrogallol. The SOD activity is directly correlated to the occurred inhibition in the investigated samples.

### 4.8. Determination of SIRT-1 and Nf-ķB Using Western Blot Analysis

The hepatic tissues were subjected to homogenization in 1 mL TriFast buffer (Peqlab, VWR company, Darmstadt, Germany) at 4 °C for 5–15 min, followed by centrifugation at a maximum of 12,000× *g* max for 10 min. The Bradford method was used to determine the concentration of the protein fraction. Equal protein content (30 μg) was loaded into each lane to be separated using sodium dodecyl sulphate-polyacrylamide gel electrophoresis. HybondTM nylon membrane (GE Healthcare, Chicago, IL, USA) served as the solid support for the proteins after transfer. A Tris buffer solution containing 5% non-fat dry milk was used for blocking. The membranes were treated with it for 60 min at ambient temperature. Then, the membrane was incubated overnight at 4 °C in antibody solution targeting NF-κB (catalog# 8242, abcam, Cambridge, MA, USA) and SIRT-1 (catalog# 110304, abcam, Cambridge, MA, USA). Afterwards, the membranes were incubated with the horseradish peroxidase (HRP)-conjugated secondary antibody for 1 h at room temperature. Visualization of protein bands was carried out by the chemiluminescence method, and the appeared bands were analyzed using ImageJ^®^ software, version 1.54r (National Institutes of Health, Bethesda, MD, USA) regarding the bands of the loaded control β-actin and calibrated as fold-change values from the control.

### 4.9. Determination of TNF-α, IL-6, pERK, and Caspase-3 Using ELISA Technique

A commercially available IL-6 (catalog# E-EL-R0015, Elabscience Biotechnology, Inc., Houston, TX, USA), TNF-α (catalog# E-EL-R0019, Elabscience Biotechnology, Inc., Houston, TX, USA), p-ERK (catalog# ELK9246, ELK biotechnology, Sugar Land, TX, USA), and caspase-3 (SL 1366Ra, Sunlong Biotech Co., Hangzhou, China) ELISA kits for rats were used, which operate on the Sandwich ELISA principle. The kit included a microtiter plate that had been pre-coated with a biotin-conjugated antibody designed to specifically bind to TNF-α, IL-6, p-ERK, or caspase-3. After incubation with an avidin-HRP conjugate and a biotinylated antibody, a tetramethylbenzidine substrate solution was added. A color change occurred in wells containing TNF-α, IL-6, p-ERK, or caspase-3, along with the biotin-conjugated antibody and enzyme-conjugated avidin. The reaction was terminated by the addition of sulfuric acid, after which the absorbance was then measured using a spectrophotometer at 450 nm. The concentrations of TNF-α, IL-6, p-ERK, and caspase-3 were quantified by comparing the optical density of the samples to a standard curve, which directly correlated with their concentrations.

### 4.10. Determination of ERK and Caspase-3 Using qRT-PCR

Real-time PCR was conducted to assess the mRNA expression of target genes. TRIzol™ reagent (Life Technologies, Carlsbad, CA, USA) was used to extract the total RNA from the tissue samples, with the procedure being completed in an hour. A total of 1 μg of extracted RNA was reverse transcribed into cDNA utilizing the validated QuantiTect Reverse Transcription Kit (Qiagen, Germantown, MD, USA) with a random hexamer primer, in accordance with the manufacturer’s protocol, guaranteeing high efficiency and minimal inhibitor interference. The cDNA samples were then analyzed for *ERK* and *caspase-3*. β-actin, a housekeeping gene, served as an internal control, and a non-template control (water) was included to verify the absence of DNA contamination in the reaction mixture. RT-PCR data were analyzed using the 2^−ΔΔCt^ method for relative gene expression quantification. The sequences of used primers are shown in Table 2.

All data collection and normalization were performed using the Rotor-Gene Q system (Qiagen, Germantown, MD, USA), with Ct values normalized to the average of the housekeeping gene β-actin. The stability of β-actin across all treatment groups was experimentally verified to ensure reliable normalization. In addition, PCR efficiency for all primer pairs, including β-actin, was confirmed by standard curve analysis to fall within the acceptable range of 90–110%, ensuring the overall accuracy and validity of the gene expression data.

### 4.11. Statistical Analysis

Data are expressed as mean ± SEM. Statistical analysis was performed using one-way ANOVA, followed by Tukey–Kramer post hoc testing to compare groups. Calculations were carried out with GraphPad Prism software (version 5.0.2; San Diego, CA, USA), and a *p* < 0.05 was considered statistically significant.

## 5. Conclusions

In conclusion, this research highlights the substantial protective role of lutein in dampening MTX-induced hepatic damage. Modulation of SIRT1, antagonizing oxidative damage and suppressing both inflammatory (NF-κB/TNF-α/IL-6) and apoptotic (ERK/caspase-3) pathways, justifies the lutein-produced improvement in hepatic function and preservation of liver structure. For lutein validation clinically, there is a demand for further clinical studies.

## Figures and Tables

**Table 1:** Effect of lutein (50 and 100 mg/kg/day, I.G.) on relative hepatic weight, serum ALT, and AST in a rodent model of MTX-induced hepatotoxicity.

Groups	Relative Hepatic Weight	Serum ALT (U/L)	Serum AST (U/L)
Control	2.92 ± 0.16	33.27 ± 1.08	39.53 ± 0.77
Lutein 100 mg	3.00 ± 0.16	33.50 ± 1.01	48.95 ± 2.14
MTX	3.12 ± 0.10	77.88 ± 2.29 *	156.1 ± 10.34 *
MTX + Lutein 50 mg	2.97 ± 0.12	50.55 ± 2.17 #	88.25 ± 7.63 #
MTX + Lutein 100 mg	2.90 ± 0.13	37.41 ± 3.64 #@	43.90 ± 2.47 #@

All data points are shown as the means ± SEM (*n* = 6–8). * Significant different compared to the control group, # significant different compared to the MTX group, and @ significant different compared to the MTX + Lutein 50 mg group at *p* < 0.05. MTX: Methotrexate, ALT: Alanine aminotransferase, AST: Aspartate aminotransferase.

**Table 2:** List of primer sequences.

Gene	Sequences (5′-3′)	
	Forwarded	Reverse
*ERK*	CCCTCTAAAACCAA GGTGGCT	CATCCAATCACCCACACAC AG
*Caspase-3*	TGAGCATTGA CACAATACAC	AAGCCGAA ACTCTTCATC
*β-actin*	ACCCACACTGTGCCC ATCTATG	AGAGTACTTGCGCTCAGGAG GA

## Data Availability

The original contributions presented in this study are included in the article. Further inquiries can be directed to the corresponding authors.

## Abbreviations

The following abbreviations are used in this manuscript:

MTX	Methotrexate
DHFR	Dihydrofolate reductase
FPGS	Folylpolyglutamate synthetase
MTX-PGs	MTX polyglutamates
SIRT-1	Sirtuin-1
TNF-α	Tumor necrosis factor-α
NF-κB	Nuclear factor kappa-B
IL-6	Interleukin-6
Nrf2	Nuclear factor erythroid 2-related factor 2
NAFLD	Non-alcoholic fatty liver disease
ROS	Reactive oxygen species
ALT	Alanine aminotransferase
AST	Aspartate aminotransferase
H and E	Hematoxylin and eosin
MDA	Malondialdehyde
GSH	Reduced glutathione
SOD	Superoxide dismutase
I.G.	Intragastric
ERK	Extracellular signal-regulated kinases
qRT-PCR	Quantitative real time polymerase chain reaction
Bax	Bcl-2 associated X protein
Bcl-2	B-cell lymphoma 2
MAPK	Mitogen-activated protein kinase
I.P.	Intraperitoneal
